# Models of Trachoma Transmission and Their Policy Implications: From Control to Elimination

**DOI:** 10.1093/cid/ciy004

**Published:** 2018-06-01

**Authors:** Thomas M Lietman, Amy Pinsent, Fengchen Liu, Michael Deiner, T Deirdre Hollingsworth, Travis C Porco

**Affiliations:** 1 Francis I. Proctor Foundation, San Francisco; 2 Department of Ophthalmology, San Francisco; 3 Department of Epidemiology and Biostatistics, San Francisco; 4 Global Health Sciences, University of California, San Francisco; 5 School of Public Health and Preventative Medicine, Monash University, Melbourne, Australia; 6 Big Data Institute, Li Ka Shing Centre for Health Information and Discovery, University of Oxford, United Kingdom

**Keywords:** trachoma, *Chlamydia trachomatis*, WHO 2020 Goals, transmission models, mass drug administration

## Abstract

Despite great progress in eliminating trachoma from the majority of worldwide districts, trachoma control seems to have stalled in some endemic districts. Can mathematical models help suggest the way forward? We review specific achievements of models in trachoma control in the past. Models showed that, even with incomplete coverage, mass drug administration could eliminate disease through a spillover effect, somewhat analogous to how incomplete vaccine campaigns can eliminate disease through herd protection. Models also suggest that elimination can always be achieved if enough people are treated often enough with an effective enough drug. Other models supported the idea that targeting ages at highest risk or continued improvements in hygiene and sanitation can contribute meaningfully to trachoma control. Models of intensive targeting of a core group may point the way to final eradication even in areas with substantial transmission and within-community heterogeneity.

In 1999, the World Health Organization (WHO) declared a goal of reducing infectious trachoma to a level such that the potential for blindness resulting from trachomatous infections would no longer be a public health concern [1]. Specifically, WHO aimed to reduce the prevalence of the clinical signs of active trachoma (follicular trachoma [TF]) to <5% in all endemic districts worldwide. To achieve this, WHO proposed annual mass drug administration (MDA) worldwide to all endemic populations, targeting the ocular strains of *Chlamydia trachomatis*, the bacterium that causes trachoma [2]. Recommendations included 3–5 annual mass antibiotic distributions to at least 80% of the population (a single dose of oral azithromycin, or 6 weeks of topical tetracycline for infants for whom oral azithromycin is not appropriate). In addition, facial hygiene and environmental (F&E) programs were recommended as a more sustainable way to reduce transmission [3]. These F&E initiatives were designed to encourage face washing and latrine building. Latrine building is intended to limit the preferred breeding ground of *Musca sorbens*, a presumptive mechanical vector of *C. trachomatis* [4].

Following nearly 2 decades of this strategy, the disease burden of trachoma has been substantially reduced, with a reported 2016 worldwide prevalence of 3338000 (Uncertainty Interval, 2439000–4492000) [5]. Control programs have achieved remarkable success in the vast majority of districts worldwide [2]. Elimination has recently been successfully achieved in countries such as Morocco, Laos, Nepal, and Mexico [6–10]. However, some severely affected districts in Ethiopia have been treated for 10 years, and are still not close to reaching the WHO-specified threshold for declaring control and halting MDA. Mathematical models of disease transmission may have helped to provide some initial rationale for the frequency of MDA treatments in typical endemic regions that were successfully treated. Now mathematical and statistical models can be used to evaluate the impact of potential alternative interventions, treatment strategies for the remaining high-prevalence areas, and to forecast if and when control will be achieved worldwide. In this article, we review some of the ways mathematical and statistical models have been used over the past 2 decades to predict future prevalence and to explore the impact of different control interventions and predict disease prevalence.

## FORECASTING OF PREVALENCE FOR PROGRAMMATIC DECISION MAKING

The programmatic outcome of an MDA campaign is typically evaluated using district-level prevalence surveys. Trachoma forecasts have been generated using a number of different approaches, including expert opinion, mechanistic models that include knowledge of disease transmission, and statistical models that include no specific knowledge of trachoma. One study demonstrated that both statistical and mechanistic models perform far better than expert opinion at forecasting trachoma prevalence 1 year in advance [11–13]. A separate analysis suggested that forecasts generated by more complex mechanistic models do not perform better than forecasts generated by purely statistical models [11]. However, even the statistical models are not that accurate at predicting individual district-level prevalence. This is in part because surveys are relatively infrequent, and in part because of considerable fluctuations in TF prevalence observed from year to year within a single district. This large apparent stochastic variation may mean that any improvement in prediction offered by additional surveys may be limited.

**Figure 1.**
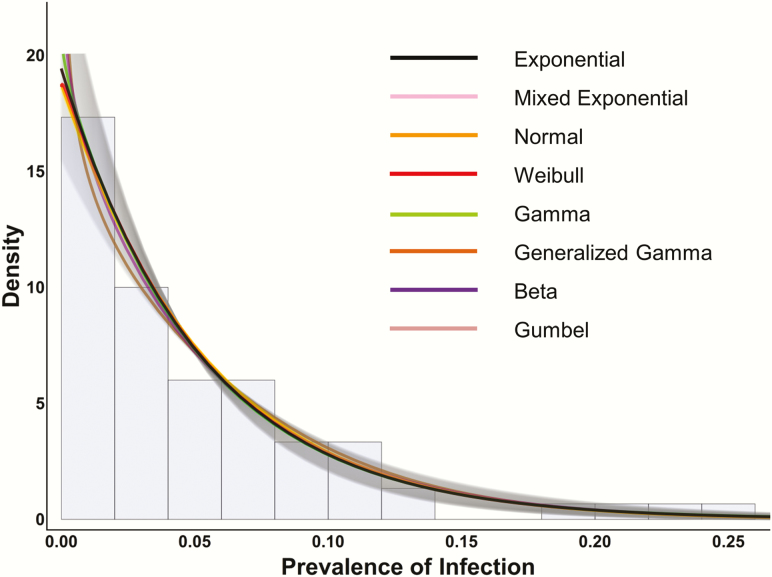
Distribution of community-level trachoma prevalence after multiple mass drug administrations. This figure shows the Tanzanian trachoma prevalence data as a histogram of district-level prevalences in the background, normalized so that the total area of the bars adds up to 100%. The best fit to the data was provided by an exponential distribution and is shown in black, along with the 95% confidence interval (CI) as gray shading. The other distributions shown (normal, Weibull, gamma, generalized gamma, beta, Gumbel, and a mixture of 2 exponential distributions) are multiple parameter distributions that include the exponential distribution as a special case. For fitting to the Tanzanian prevalence data, these more complex distributions clearly mimic the exponential with their fit within the 95% CI of the exponential. This suggests that distributions more complicated than the exponential are not necessary to describe the data. Reprinted with permission from Rahman et al [15].

On the other hand, forecasting changes in the distribution of district-level prevalence across a group of districts allows the model to leverage the information across multiple, highly stochastic processes, and has proven surprisingly successful (T.M.L., unpublished data).The distribution of the prevalence of trachoma across many districts can even be used to identify whether or not control is failing. Theoretical and empirical studies suggest that under subcritical conditions, the distribution of district-level prevalence should approach a geometric or exponential distribution. In Figure 1, the distribution of infection from 75 communities in Tanzania is plotted in a histogram, and fitted well by an exponential distribution [14–16]. The fact that the distribution worldwide became approximately exponential in 2010 suggests that trachoma programs may have achieved control in most areas (T.M.L., unpublished data). Unfortunately, a closer examination reveals some outliers, unlikely to have come from an exponential distribution. The departure from exponential suggests that more will need to be done to control trachoma in these areas, which are for the most part in a few severely affected regions of Ethiopia (T.M.L., unpublished data). Here models could help provide insight into what intervention strategies could be implemented to help reach control.

Traditional definitions of hypo-, meso-, and hyperendemic regions based on baseline prevalence of TF have been useful to trachoma programs. However, because of the stochasticity mentioned above, it can be challenging to know how to interpret a single community with high prevalence (eg, 20%) within an area with generally lower prevalence (eg, a mean of 8%). This would be expected given the relatively heavy tail of the exponential distribution, and would not in itself indicate program failure or a hot spot that required more resources. Such a prevalence is completely consistent with expected fluctuations under subcritical conditions. On the other hand, in a region with multiple high-prevalence districts where the prevalence is not distributed exponentially, more resources may be needed to achieve control. One of the interesting questions is at what level (subnational, international) models are most effective at identifying hotspots and in offering reassurance on program progress.

## THE EFFECT OF EXISTING AND NEW STRATEGIES, INFORMED BY MODELING

Forecasts suggest that achievement of the global goals in all endemic districts will not be achieved by 2020, with several regions (essentially in Ethiopia) as clear outliers. Empirical evidence confirms that this is not solely due to a late initiation of treatment programs. The question then arises of what is the correct strategy in areas where the target is not being met. The simplest strategic response is simply to treat for more years. However, a plausible hypothesis is that transmission is simply so strong in these areas that current strategies are not sufficient to control disease. In these areas, mathematical models can suggest novel strategies that would then be testable in practice.

## FACTORS THAT MAY UNDERMINE THE SUCCESS OF AN MDA PROGRAM

**Figure 2.**
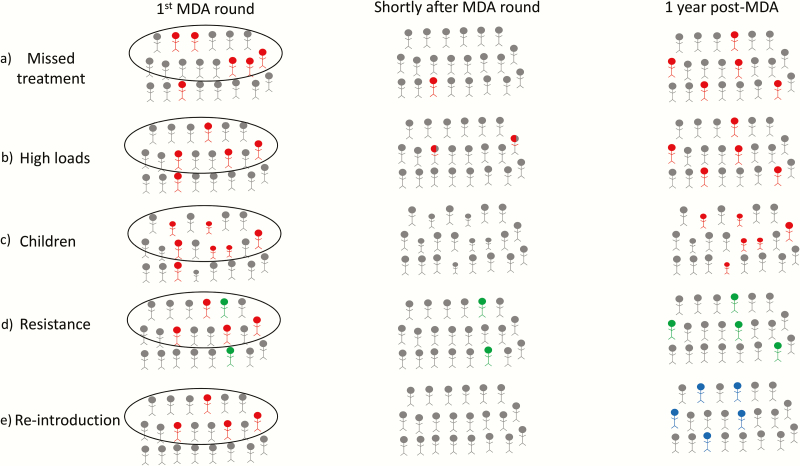
Schematic illustrating the potential reasons for failure of mass drug administration (MDA) within a community. *A*, Some infected individuals (in red) within the community miss treatment and act as a reservoir source of infection to the rest of the community. *B*, Infected individuals with high bacterial loads who are treated do not fully resolve their infection (in red) following 1 dose of treatment post-MDA and thus remain a source of infection to the community. *C*, Children make up the primary source of infections to the community and, following MDA, infection is most likely to return in children (red). *D*, Individuals infected in the community (red) are treated, but some individuals are infected with a resistant strain (green) and therefore treatment is ineffective; thus, individuals infected with the resistant strain can reseed infection into the community following MDA. *E*, Infected individuals in the community (red) are treated effectively following MDA; the community remains infection free for a period but infection is reintroduced from a neighboring community (blue).

In some severely affected areas, 10 years of annual MDA has not come close to achieving the WHO threshold for control, with lingering infection within a community. Several hypotheses may explain the persistence of residual infection (Figure 2). Systematic noncompliance (a group of individuals who are systematically not accessing or seeking MDA treatment) may prevent success, if sustained transmission is achieved in this group [17] (Figure 2A). More data on noncompliance, from coverage surveys or other tools, could be useful in evaluating this possibility [17, 18]. The practical importance of noncompliance depends on which age group drives transmission. Modeling suggests that given the high prevalence of infection in 1- to 5-year-olds, intensive targeting of children could still in principle be sufficient to overcome a degree of noncompliance in adults that was correlated over time [19] (Figure 2C).

Alternatively, individuals with high-bacterial-load infections may have a poorer chance of fully clearing the infection with a single dose of antibiotic treatment [20] (Figure 2B). Modeling suggests that if this could be overcome by 2 treatments within 2 weeks of each other, the number of “double-dose” annual campaigns would be reduced [21]. This model assumed that the efficacy of the 2 doses were not independent, with the second dose being more effective (further discussed below).

It is also possible that successive rounds of treatment may have selected for macrolide-resistant strains of chlamydia. This has not been found to be the case in the few studies that have assessed chlamydial resistance [22–24] (Figure 2D). Modeling has offered some insight into the regression of macrolide resistance in *Streptococcus pneumoniae* after MDA has been discontinued [25]. Macrolide resistance in *C. trachomatis* has not yet been found empirically, but the possibility of resistance could be modeled in the future. Alternatively, reintroduction from outside the community may be occurring (Figure 2E).

It is unlikely that any one of these possible mechanisms is the sole cause of the persistence of residual infection. Accordingly, efforts to intensify MDA and to conduct activities in addition to MDA may be needed.

## INTENSIFICATION AND OPTIMIZATION OF ANTIBIOTIC CAMPAIGNS

Repeated MDA appears to confer indirect protection to untreated individuals, a spillover effect analogous to herd protection in vaccines [26]. Mathematical modeling has suggested that infection can be eliminated from the most severely affected areas even without complete coverage, and this has been confirmed by longitudinal studies and community randomized trials. In these models, the specific frequency and coverage of treatment is important to success [27, 28].

## INCREASING THE FREQUENCY OF ANTIBIOTIC DISTRIBUTIONS

**Figure 3.**
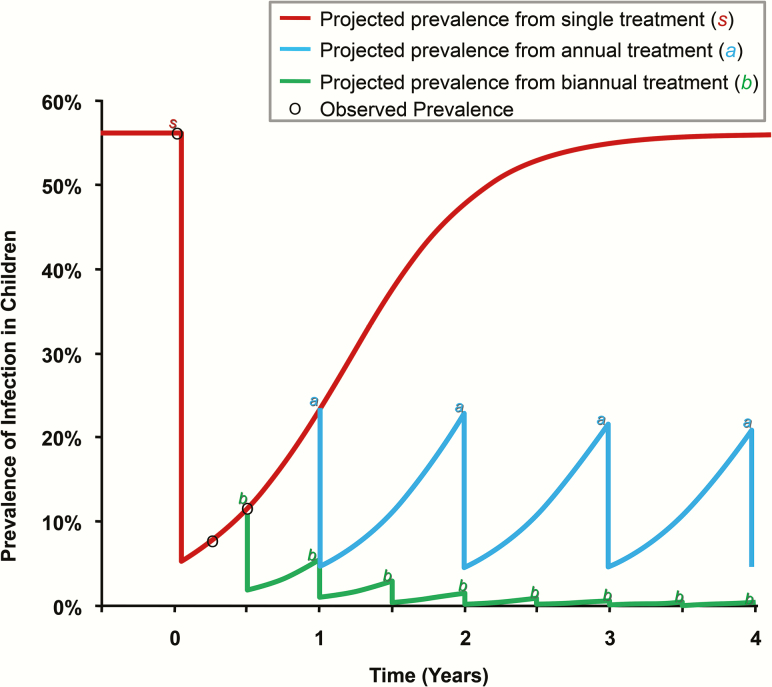
Frequency of mass drug administration necessary to achieve elimination. Projections are based on the assumptions that treatment is administered once, annually, or biannually; coverage is 80%; and antibiotic efficacy in an individual is 95%. Projections follow logistic growth determined by the following equation: baseline prevalence / (1 + *C e*^−[rate of return][time]^), where *C* is a constant that is fit by the empirical data. Adapted from Melese et al [28].

Transmission modeling analyses originally suggested that in hyperendemic areas, annual MDA with approximately 80% coverage might not be sufficient to eliminate infection, and that biannual treatment would be required in these areas (Figure 3) [27, 28]. Three clinical trials revealed that biannual treatment may eliminate infection somewhat more rapidly than annual treatment, although no significant differences could be found after 3–4 years of treatments [29, 30].

## INCREASING ANTIBIOTIC COVERAGE

Mathematical models suggest that attaining a high level of coverage when implementing MDA is as important for elimination as the frequency of antibiotic distribution and efficacy of treatment [27]. However, programmatic MDA coverage is notoriously difficult to assess in practice. It is difficult to know how many individuals in a community were not identified in the pretreatment census, and hence accurately quantify the denominator population that required treatment. A set of 3 community-randomized trials addressed antibiotic coverage in hypo- and mesoendemic areas (Partnership for Rapid Elimination of Trachoma [PRET]). Communities were randomized to 80% coverage (a level recommended by the WHO and achievable in a single day) vs >90% coverage (only attained after 1–3 follow-up visits). Investigators were unable to demonstrate a statistically significant improvement with enhanced coverage in any of the 3 country settings [31, 32]. Note that this did not study the range of lower coverages that may be present in some distribution programs.

## OPTIMIZING THE TIMING OF ANTIBIOTIC DISTRIBUTION

Clinically active trachoma (TF) has been noted to vary over the course of the year, although definitive seasonality in infection has been more difficult to demonstrate [33]. If seasonal variation in transmission were pronounced, one mathematical modeling study suggested that treatment 3 months before the high transmission season might be the most effective [34]. In areas where transmission is constant throughout the year, mathematical models have looked at the optimal timing of a second treatment: either soon after the first (eg, 1–2 weeks after [21]), or 6 months apart. If 2 treatments closer together result in the second treatment being more effective, then short-interval double treatment results in higher clearance and reduced transmission [21]. If, however, treatment efficacy and coverage at each time point were independent, then it is straightforward to demonstrate that the timing of the second treatment does not matter [35]. The Azithromycin in the Control of Trachoma (ACT) trial found remaining infection in the community after 3 weekly treatments with >95% coverage, suggesting that a second or third weekly dose may not result in a dramatic improvement in prevalence [36]. Individual randomized trials comparing different regimens have had difficulty finding significant improvements [37, 38]. A trial that assesses a second weekly dose is currently being set up (The Carter Center – Trachoma Elimination Study by Focused Antibiotics [TCC-TESFA]), as is another evaluating 2 doses 2 weeks apart.

## TARGETING CORE GROUPS

**Figure 4.**
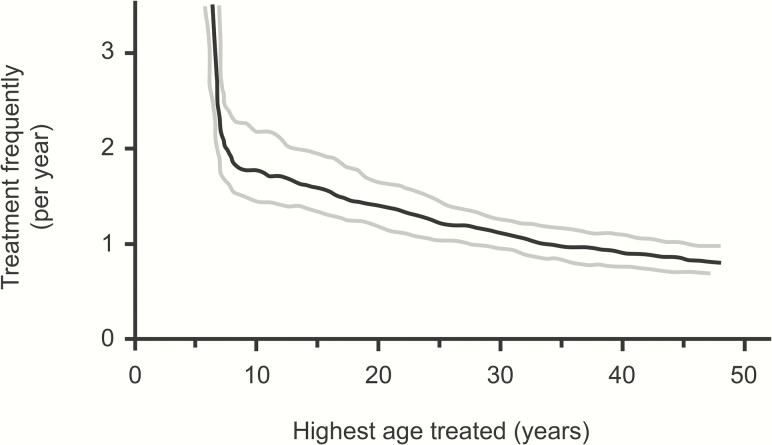
The necessary frequency of treatments that will ensure the eventual elimination of active trachoma if only individuals under a certain age are treated. Although annual treatment may well eliminate disease if everyone in the population is treated, biannual treatment may be necessary if treatment is limited to children 1–10 years old. Results of the uncertainty analysis: black curve, 50th percentile; gray curves, 25th and 75th percentiles (based on data from Malawi). Adapted from Lietman et al [27].

The largest risk factor for infection is age, with prevalence most commonly peaking in preschool children. Children are thought to have a longer duration of infection, and higher bacterial loads. If these highly infectious children could be removed from the transmission network, infection could eventually disappear in the community (Figures 2C and 4). One mathematical modeling study suggested that frequent treatment of children alone could eventually eliminate infection in the entire community, and short-term trial results are consistent with this [19]. A community-randomized trial of treatment directed to children 4 times in a year in Ethiopia found that the prevalence of infection in untreated adults was reduced nearly in half by the end of the year [26], and quarterly treatment of children was superior to annual treatment of everyone in one trial. Biannual treatment of children aged 0–12 years in Niger also reduced infection in adults, and had similar efficacy to treating all ages once per year [39].

Treatment of clinically active children and their households has had modest success in community-randomized trials in Nepal and Ethiopia, although an analysis including some transmission modeling suggested that it may not be cost-effective due to the cost of identifying actively affected children [40–42] (National Institutes of Health [NIH], Trachoma Amelioration in Northern Amhara II [TANA II] study).

## TREATMENT OF A CORE GROUP REMAINING INFECTED DESPITE MANY ROUNDS OF MDA

Individuals in the community who are infected at an endemic equilibrium may represent a possible core group for the transmission of infection. In a sense, communities in the most severely affected areas have reached a new “steady” state, or equilibrium, where prevalence remains constant at the new lower level after 5–10 years of MDA. While this level may be far lower than the pretreatment prevalence, this new equilibrium can still be higher than control targets of <5% TF. Epidemiological studies have shown that those currently infected with ocular chlamydia are the most likely to be infected later, even if after being cleared of their original infection [43–45]. Thus, the group that remains infected may form a residual core group—if annual distributions were continued *and* this group was effectively taken out of the transmission network, mathematical models suggest that infection could be completely eliminated [27]. If these groups could be identified, quarterly treatment of this group with oral azithromycin could effectively remove them from the transmission network. Although resource intensive, this strategy may be what is required in a small number of severely affected communities where infection continues to persist. Two versions of the strategy targeting a residual core group are at different stages of clinical trial testing (NIH studies Sanitation, Water, and Instruction in Face-washing for Trachoma [SWIFT] and Kebele Elimination of Trachoma For Ocular Health [KETFO]), although larger-scale studies may be required.

## INTENSIFY THE HYGIENE AND SANITATION COMPONENTS

Trachoma disappeared from some areas of the world before the discovery of antibiotics, which has led many to believe that improved environmental conditions may help to reduce transmission of infection [46]. Mathematical modeling has demonstrated that *if* reductions in the transmission potential of the pathogen could be achieved by improving water supply, sanitation, and hygiene (WASH), then control could be more achievable and sustainable in certain locations [21]. Unfortunately, no additional benefit from F&E interventions has ever been proven. While WASH indicators clearly correlate with a lower prevalence of trachoma at the individual and community level [47], no clinical trial has demonstrated that any nonantibiotic intervention actually reduces chlamydial infection [3, 48]. A large community-randomized trial is now being conducted to assess whether intensive F&E reduces infection in a severely affected area (NIH National Eye Institute, SWIFT), and other trials are also being planned to test the hypothesis. If the trials can prove that any particular F&E intervention actually reduces infection, this could be incorporated into future models [21, 49].

## CONCLUSIONS

In regions where the WHO trachoma strategy is currently not controlling the disease, mathematical models can be used to suggest, evaluate, and investigate the possible impact of alternative strategies. These can then be tested in a clinical trials setting in a falsifiable manner. Increased dosing at the individual or the community level and the impact of F&E continue to be assessed in large randomized trials. If an effect is demonstrated, it might facilitate elimination in currently difficult-to-treat areas. Intensive targeting of a residual core group that remains infected following annual MDA is currently being tested in severely affected regions of Ethiopia. While transmission models may help inform the design of new strategies for these regions, a number of biological uncertainties underlying these models remain, and more detailed data would probably be required to distinguish between them [50]. Advances in mathematical and statistical modeling are allowing forecasting of the district-level prevalence of trachoma to be improved. While statistical forecasting suggests that control worldwide in 2020 is extremely unlikely, success in the next decade is indeed feasible, and forecasts can help with planning and with keeping expectations realistic.

## Notes

***Disclaimer.*** The views, opinions, assumptions or any other information set out in this article are solely those of the authors and should not be attributed to the funders or any person connected with the funders.

***Financial support.*** This work was supported by the Bill & Melinda Gates Foundation in partnership with the Task Force for Global Health through the Neglected Tropical Diseases Modelling Consortium (grant number OPP1053230); National Eye Institute (NEI) (R01 grant number EY025350 to the University of California, San Francisco [UCSF]); Research to Prevent Blindness (unrestricted grant to UCSF); and NEI Core Grant (number EY002162 to UCSF).

***Supplement sponsorship.*** This article appears as part of the supplement “Reaching the 2020 Goals for Nine Neglected Tropical Diseases,” sponsored by the NTD Modelling Consortium.

***Potential conflicts of interest.*** All authors: No reported conflicts of interest. All authors have submitted the ICMJE Form for Disclosure of Potential Conflicts of Interest. Conflicts that the editors consider relevant to the content of the manuscript have been disclosed.
